# Delayed entry into HIV care after diagnosis in two specialized care and treatment centres in Cameroon: the influence of CD4 count and WHO staging

**DOI:** 10.1186/s12889-016-3258-8

**Published:** 2016-07-08

**Authors:** Noah F. Takah, George Awungafac, Leopold N. Aminde, Innocent Ali, Juliana Ndasi, Patrick Njukeng

**Affiliations:** Global Health Systems Solutions, Limbe, Cameroon; Faculty of Health Sciences, University of Buea, Buea, Cameroon; Sub divisional Hospital Nguti, Nguti, South West Region Cameroon; Clinical Research Education, Networking and Consultancy, Douala, Cameroon; Virology Laboratory, University of Dschang, Dschang, Cameroon

**Keywords:** Delayed entry, Factors associated

## Abstract

**Background:**

Delayed entry into HIV care has complicated the challenges faced in sub-Saharan Africa due to the high HIV burden. A clear knowledge of the factors affecting delayed entry will be essential in directing interventions towards reducing delayed entry into HIV care. There exist very limited data on delayed entry in Cameroon despite its relevance; hence this study was conducted to determine the rate of delayed entry and its associated factors in HIV programmes in Cameroon.

**Methods:**

Data used for this study was routine data obtained from the files of HIV patients who were diagnosed between January 1, 2015 and June 30, 2015 at Limbe and Buea regional hospital HIV centers in the South West region of Cameroon. Data analysis was done using SPSS version 20.

**Results:**

Of the 223 patients included in the study, nearly one-quarter of patients (22.4 %) delayed to enter HIV care within 3 months. Those who delayed to enter care were less likely to present at first diagnosis (using HIV rapid test) with symptoms such as fever > 1 month (5 % versus 30 %, *p* = 0.01) and weight loss > 10 % (13 % versus 48 %, *p* < 0.001). Alcohol consumption, WHO stage and CD4 count levels were also associated with delayed entry in bivariate analysis. In multivariate analysis only CD4 count greater than 500cells/μl and WHO stages I and II were independently associated with delayed entry into HIV care within 3 months.

**Conclusion:**

In the South West region of Cameroon, approximately 1 out of 4 patients delay to enter HIV care. This high proportion of patients who delay to enter care correlates to the findings recorded by other studies in sub Saharan Africa. Interventions tackling delayed entry into HIV care might need to be favorably directed towards patients that have high CD4 counts and are at very early WHO clinical stages.

## Background

The global burden of HIV is clearly unevenly distributed with sub-Saharan Africa having the greatest share of the spread and sequelae of the pandemic [1, 2]. With an estimated 24.7 million people living with HIV in sub-Saharan Africa, accounting for almost 71 % of the global HIV burden, the region continues to face enormous challenges to control the spread of the pandemic as well as to adequately and effectively managed old and newly infected people living with HIV [3]. Despite the successes registered so far in the domain of increasing access to antiretroviral medications and curbing the spread of new HIV infections, the effective tracking and monitoring of HIV infected patients still present as daunting challenges that urgently need to be addressed for the success of HIV care programmes in the sub-Saharan Africa region [4–10].

All across Africa (sub-Saharan Africa and Cameroon in particular),the United States (US) President’s Emergency Plan For AIDS Relief (PEPFAR) through the US Centres for Disease Control and Prevention (CDC) have directed substantial amounts of funds towards addressing the challenges faced by countries towards HIV diagnosis [11]. Through local implementing partners, the efforts of PEPFAR and the CDC have resulted in improved CD4 testing that ensures the issuing of same day CD4 test results to newly diagnosed HIV positive patients [12, 13]. Despite these efforts, several studies in Africa have reported delayed entry into HIV care and loss to follow up after initial HIV diagnosis [14–16]. Delayed entry into HIV care has been defined by the World Health Organization [17] and from several published studies as not having a CD4 measurement within 3 months of HIV diagnosis [18–20]. A better understanding of the factors affecting delayed entry before antiretroviral therapy initiation will be necessary in guiding interventions towards ensuring timely CD4 measurement and eventual enrolment into the HIV continuum of care [21]. Despite the relevance of studies in delayed entry into HIV care, there exist limited published data on its epidemiology in Cameroon. This study was therefore designed and conducted to determine the prevalence of delayed entry into HIV care, the socio-demographic and clinical profiles of patients newly diagnosed of HIV and the factors that are associated with delayed entry into HIV care in Cameroon.

## Methods

### Study design and Area

This was a cross-sectional study conducted in the two major HIV care and treatment centres in the South West region of Cameroon namely the Buea Regional and Limbe Regional Hospitals, from the 1st January 2015 to June 30th 2015. The hospitals selected cover more than two thirds of the total HIV population in the South West region and are well situated in the Regional Capital (Buea health district with population = 133,092 inhabitants) and the Divisional Capital (Limbe health district with population = 151,258 inhabitants) with relatively high population densities. Both centres are equipped with the Alere PIMA CD4 point of care diagnostic machines permitting the issuing of same day CD4 results. Each of the centres receives averagely 20–23 newly diagnosed HIV positive patients every month.

### Sample size and sampling technique

A convenience sampling technique was used. A total of 223 adult (≥18 years) non pregnant patients who presented at both centres and were newly diagnosed HIV positive between January 1st 2015 to June 30th 2015 were included in the study consecutively.

### Data collection and study procedure

Data used for this study was routine data obtained from the files of HIV patients who were diagnosed between January 1, 2015 and June 30, 2015 at Limbe and Buea regional hospitals HIV centers in the South West region of Cameroon. A structured data collection form was developed by the research team following the flow of HIV programme activities from patient identification, consultation, pre-test counselling and testing and post-test counselling in the two centres. The data collection form consisted of socio-demographic characteristics, the medical history of the patients, the clinical presentation and WHO staging, the laboratory tests and the date when HIV rapid test and CD4 test were done. The socio-demographic characteristics included the age, sex, religion (Christian, Muslim and others), marital status, educational level, household size, sharing of results, occupation, place of residence and time taken to reach treatment centres. The educational level was classified based on the level of study and/or certificates obtained. The educational levels were classified into primary (if elementary school was attended and/or first school living certificate was obtained), secondary (if college was attended and/or college certificates obtained) and university education (if a school of higher learning was attended and/or certificates obtained). The number of family members in each household (household size) was recorded. The time taken to reach the treatment centres was classified into less than 1 h and greater than 1 h. The data collection form also included questions on whether the participants shared their results with close family members (wife, children, siblings or parents) or friends by showing the HIV test result to them. In the medical history, presence of other associated chronic diseases (Tuberculosis, diabetes, hypertension and hepatitis) and sexually transmitted infections was noted. Alcohol consumers were classified into low, moderate and excessive alcohol consumers. Excessive alcohol consumption was based on intake of either more than 3 (2 for women) standard glasses of wine per day or more than 10 (5 for women) local beers (1 local beer contains 28 g of alcohol) per week. Moderate alcohol consumption was based on intake of 25–30 g per day. The clinical symptoms and signs were noted for each patient by the nurses at the treatment centre and this was used to classify the patients according to the World Health Organization (WHO) staging [22]. The laboratory tests performed by the patients were also collected and included HIV rapid tests (for HIV ½ and the time when test was performed), CD4 test (the value and the time test was performed), hemoglobin and full blood count. The drug history of the patients was also noted.

After the administrative and ethical approvals had been obtained, the investigators proceeded to complete the data collection form, using the information from the files of the patients that were initially filled by the nurses at the care and treatment centres.

### Definition of delayed entry

A patient was said to have delayed to enter HIV care if he/she did not have a CD4 measurement within 3 months of HIV diagnosis [18, 23].

### Data analysis

Data were analyzed using the Statistical Package for Social Sciences (SSPS Inc, Chicago, Illinois, USA) version 20.0 software. Results are summarized as counts and percentages for categorical variables and as mean and standard deviation (SD) for quantitative variables. Group comparison used the Student *t*-test for quantitative variables and the chi-square test for categorical variables. To assess the factors associated with delayed entry, bivariate and multivariate logistic regression models were used. A *p*-value 0.05 was considered statistically significant.

We controlled for confounding in the analysis by using a multiple logistics regression through backward elimination in which if a variable was suspected to be confounder, we removed the variable from the model and checked if the odds ratios and significance of the rest of the variables left in the new model changed significantly. If removing a variable from the model resulted in a significant change in the odds ratios, they were maintained in the model even if they were statistically insignificant.

## Results

### Clinical characteristics of the study population

In this study, the participants were between 18 and 65 years old with a median age of 32 years. 88 (39.5 %) of the participants were between 21 and 30 years old, 140 (62.8 %) of the participants were females and 90 (43.7 %) had primary education (Table 1). In addition, 178 (79.8 %) shared their results with close family members (Table 2).

**Table 1:** Socio-demographic characteristics of the study population (*N* = 223)

Characteristics	Frequency/Mean ± SD	Percentage
Age, Mean ± SD, median(range)	33.3 ± 9.3, 32 (18–65)	
Age groups		
<20	11	4.9
21–30	88	39.5
31–40	74	33.2
41–50	39	17.5
51–60	10	4.5
>60	01	0.4
Gender		
Male	83	37.2
Female	140	62.8
Religion		
Christian	198	88.8
Muslim	25	11.2
Marital status		
Married	81	36.3
Single	128	57.4
Widow(er)	14	6.3
Educational level		
Never	19	9.2
Primary	90	43.7
Secondary	73	35.4
Higher/University	24	11.7

### Prevalence of delayed entry into HIV care and its associated factors

Our findings reveal that, nearly 1 out of 4 patients (22.4 %) delayed to enter care within 3 months of initial HIV diagnosis (Table 2). Those who delayed to enter care were less likely to present for HIV rapid testing with symptoms such as fever > 1 month (5 % versus 30 %, *p* = 0.01) and weight loss > 10 % (13 % versus 48 %, *p* < 0.001) (Table 2). Alcohol consumption, WHO stage and CD4 count levels were also associated with delayed entry in bivariate analysis (Table 2 and Table 3). The presence of symptoms such as fever and weight loss seemed to be strong predictors of delayed entry (and indirectly care seeking behaviour) but further analysis revealed that they were confounded by the WHO stages I and II. In multivariate analysis, only CD4 count greater than 500cells/μl⦋3.6 (0.6 – 10.4, *p* = 0.012] and WHO stages I and II⦋5.4 (1.9 – 15.2, *p* = 0.01] were independently associated with delayed entry into HIV care within 3 months (Table 3).

**Table 2:** Characteristics of the study population according to delay entry status

Characteristics	Total (*N* = 223) *n*(%) or mean ± SD	Delayed (*N* = 50) n(%) or mean ± SD	Not delayed (*N* = 173) *n*(%) or mean ± SD	*p*-value
Age	33.3 ± 9.3	33.4 ± 11.3	33.3 ± 8.7	0.10
Gender				
Male	83 (37.2)	19 (38.0)	64 (36.9)	0.10
Female	140 (62.8)	31 (62.0)	109 (63.1)	
Result shared	178(79.8)	30(60.0)	148 (85.5)	0.92
Alcohol	121 (54.3)	34 (68.0)	87 (50.3)	0.04
WHO HIV stage				
WHO I	125 (56.1)	20 (40.0)	105 (61.0)	<0.001
WHO II	38 (17.0)	17 (34.0)	21 (12.0)	
WHO III	54 (25.2)	10 (20.0)	44 (25.0)	
WHO IV	06 (2)	3 (6.0)	04 (2.0)	
Fever >1 month	35 (15.7)	5 (10.0)	30 (17.3)	0.010
Weight loss > 10 %	47 (21.1)	23 (13.3)	24 (48.0)	<0.001
CD4 count	441.4 ± 279.3	458.2 ± 281.7	384.7 ± 266.2	0.01
Travel hours to TC	0.7 ± 1.4	0.7 ± 1.5	0.3 ± 0.6	0.082

**Table 3:** Factors associated with delay entry into HIV care within 3 months; Logistics regression analysis

Characteristics	Bivariate analysis		Multivariate analysis	
	OR (95%CI)	*p*-value	OR (95%CI)	*p*-value
Age				
>32 years	Ref			
≤32 years	1.5 (0.8 – 3.1)	0.10	2.8 (0.6 – 2.3)	0.18
Gender				
Female	Ref			
Male	3.5 (1.3 – 9.2)	0.10	1.2 (0.8 – 5.5)	0.22
Educational level				
Higher	Ref			
Nil	2.1 (0.3 – 13.8)	0.45		
Primary	1.7 (0.4 – 8.1)	0.51		
Secondary	0.5 (0.1 – 3.0)	0.43		
Alcohol				
No	Ref			
Yes	2.7 (1.9 – 7.9)	0.04	1.5 (0.5 – 4.8)	0.47
Anaemia				
Hb ≤ 9 g/dL	Ref			
Hb > 9 g/dL	2.6 (0.2 - 30.2)	0.43		
CD4count				
<499cells/μl	Ref			
≥500cells/μl	4.9 (1.4 – 17.5)	0.01	3.6 (0.6 – 10.4)	0.012
Travel hours to TC				
≤1 h	Ref			
>1 h	1.2 (0.3 – 4.5)	0.75		
WHO-HIV stage				
WHO III&IV	Ref			
WHO I & II	6.1 (2.3 – 16.2)	<0.001	5.4 (1.9 – 15.2)	0.01
Sharing of result				
No	Ref			
Yes	1.2 (0.7 – 3.4)	0.92		

In the backward elimination method of adjustment for confounders in the regression model, removal of the variables such as age, gender and alcohol consumption led to significant changes in the odds ratios of CD4 and WHO stage. These variables were therefore maintained in the model.

## Discussion

Our study reveal that the proportion of those who delayed to enter HIV care was nearly 1 out of 4 patients. This high proportion of delayed entry was driven by high CD4 count at first diagnosis and early WHO clinical stages (stages I and II).

The high percentage of those who delay to enter HIV care observed in our study is comparable to many other studies that have been carried out in other parts of the World such as the United States and Europe. Rosen and colleagues carried out a systematic review between testing and treatment initiation in sub Saharan Africa and demonstrated that the rate of delay entry between HIV diagnosis and receipt of first CD4 measurement varies between 12 % and 65 % [24]. The proportion of those who delay to enter HIV care in our study is also quite close to the 29.7 % reported by Alvarez-Uria and colleagues [25] in India and 23.2 % reported by Jennes and associates [20] in the United States. Delayed entry into care therefore cuts across both developed and developing settings of the World with its highest rate in sub Saharan Africa. This similar rates of delayed entry with non-African settings can be due to the similarity in the patterns of factors associated with delay entry such as refusal to accept HIV status in both African and non-African settings [20]. The high percentage of those who delay to enter HIV care in our setting can be linked to the fact that in the early era of rapid antiretroviral treatment (ART) scale up, much attention was directed towards initiating eligible patients on ART while those who were HIV positive and not eligible (that is those in the pre-HAART period) were not given enough attention and follow up [24]. Furthermore, the rate of delayed entry can be even higher if a single group of patients is put under investigation [26].

Several studies in Africa have attempted to investigate the factors associated with the phenomenon of delayed entry into HIV care [14, 21, 25, 27]. In our study, we investigated if factors such age, gender, educational level, alcohol consumption, travel hours from the treatment centres, sharing of results with a family member or friend,anemia,CD4 count on first diagnosis and WHO clinical staging were associated with delayed entry. From our study, high CD4 (greater than 500cells/μl) and WHO stages I and II were strongly associated with delay entry. While many studies have reported this positive relationship between high CD4 and delayed entry [15, 18, 25, 28] some other studies in Guinea-Bissau and Uganda have reported an association between low CD4 count and delayed entry [9, 29]. The relationship between high CD4 count and delay entry may be related to the observation that most of such patients feel healthy or asymptomatic (WHO stage I) [18, 29], and they may tend to think they still have some time before development of symptoms or they may live in denial, refusing to accept their HIV status. Even though our study showed that age was not significantly associated with delayed entry, there exist some evidence from other studies that young age is significantly associated with other factors which may be related to delayed entry into care such as acceptance of HIV positive status and initiation on lifelong antiretroviral therapy tend to be problematic among young HIV patients [30, 31].

Using a convenient sample and a cross sectional study design poses some limitations. Importantly, a cross sectional study design can only describe temporal relationships between factors observed and the outcome irrespective of the strength of the associations observed, and therefore only provides plausible hypotheses that can be explored using other study designs [32]. Variables such as sharing of results may change over time as participants receive appropriate post-test counselling. This could not be captured by a cross sectional study design. Using a consecutive sample in a hospital setting means our study is prone to selection bias and might not be representative of the population of HIV positive patients in Cameroon. This warrants further multicenter studies which also include individuals diagnosed in non-hospital settings such as community outreach. Using routine data captured in the files of patients led to some missing data on alcohol consumption. The quality of data collected on variables such as alcohol consumption would have been much better if face to face interviews were conducted. Due to this limitation in the quality of data collected on alcohol consumption, the association with delayed entry into HIV care should be interpreted with caution. However, in our setting where diagnosis of HIV is still associated with a substantial level of stigma, face to face interviews would have markedly resulted in a low response rate and small sample size for the study. In terms of the representativeness of our sample, since we included all HIV patients diagnosed within the period of study in the two main centres responsible for HIV care in the South West region of Cameroon, our sample could be representative of patients diagnosed within health care settings in this region of the country. With regards to data collection, despite the missing data on alcohol consumption, we encountered very little challenges on the quality of data collected on other variables because we ensured that the nurses that were in-charge of filling in and coding patient files were all trained on quality of data recording. Furthermore, it is necessary to also note that this study is the first of its kind in the subject of delayed entry in Cameroon and in an era of greater emphasis on testing, treating, tracking and follow up of HIV patients, its relevance cannot be overemphasized. Our findings provide some key information that would guide interventions to address delayed entry into HIV care after diagnosis by suggesting the group of newly diagnosed HIV patients to be suitably targeted in our setting; with the goal of reducing cascade losses at a key point in the HIV care continuum. Consequently, a larger multicenter cohort study is urgently needed to fine tune our observations made so far by establishing with a higher degree of certainty the predictors of delayed entry in our setting.

## Conclusion

In the South West region of Cameroon, approximately 1 out of 4 patients delay to enter HIV care. This high proportion of patients who delay to enter care correlates to the findings recorded by other studies in sub Saharan Africa. Interventions tackling delay entry into HIV care might need to be favorably directed towards patients that have high CD4 counts and are at very early WHO clinical stages.

## Abbreviations

CDC, centres for disease control and prevention; HIV, human immunodeficiency virus; PEPFAR, president’s emergency plan for AIDS relief; WHO, World Health Organization.
